# Bilateral Tension Pneumothorax Following Unilateral Endobronchial Valve Placement: A Case Report

**DOI:** 10.7759/cureus.103187

**Published:** 2026-02-08

**Authors:** Ines Pinto Pereira, Ana Salgado, Vasco Silva, Sérgio Campainha, Susana Ferreira

**Affiliations:** 1 Intensive Care Unit, Unidade Local de Saúde Gaia/Espinho, Vila Nova de Gaia, PRT; 2 Pulmonology, Unidade Local de Saúde Gaia/Espinho, Vila Nova de Gaia, PRT

**Keywords:** bilateral tension pneumothorax, bronchoscopic lung volume reduction, endobronchial valves, point-of-care ultrasound, pulmonary critical care, severe emphysema

## Abstract

Endobronchial valves (EBVs) are increasingly used as a minimally invasive treatment option for selected patients with severe emphysema and hyperinflation. Pneumothorax is a recognized complication of EBV placement, typically occurring ipsilateral to the treated lung. To our knowledge, bilateral tension pneumothorax is exceedingly rare and potentially life-threatening.

We report the case of a 61-year-old man with severe emphysema and hyperinflation who was admitted to the intensive care unit for monitoring after unilateral EBV placement in the right upper lobe. On the second day of admission, while breathing spontaneously, he developed sudden severe respiratory distress and hemodynamic instability. Bedside thoracic ultrasound, performed immediately during equipment preparation and prior to definitive imaging, demonstrated bilateral absence of lung sliding, supporting the diagnosis of bilateral tension pneumothorax. Immediate bilateral needle decompression was followed by chest tube insertion. The patient experienced cardiac arrest with pulseless electrical activity but achieved return of spontaneous circulation after prompt intervention. A persistent high-output right-sided air leak remained despite five days of spontaneous ventilation, necessitating valve removal and chemical pleurodesis, with complete resolution of the pneumothorax.

This case illustrates a rare and catastrophic complication of unilateral EBV placement, likely related to abrupt post-procedural changes in lung mechanics and stress redistribution in severely emphysematous lungs. Early recognition, rapid bilateral decompression, and the adjunctive use of bedside thoracic ultrasound were critical for patient survival.

Bilateral tension pneumothorax, although rare, should be considered in patients with acute respiratory or hemodynamic deterioration after EBV placement. Prompt diagnosis, supported by point-of-care ultrasound, and decisive management are essential. Persistent air leaks may require valve removal and pleurodesis, highlighting the need for individualized therapeutic strategies in this high-risk population.

## Introduction

Endobronchial valves (EBVs) are one-way bronchoscopic devices used in the management of selected pulmonary conditions, particularly severe emphysema with hyperinflation. By preventing inspiratory airflow into targeted lung segments while allowing expiratory gas egress, EBVs promote lobar atelectasis and reduce dynamic hyperinflation, leading to improved respiratory mechanics and functional capacity in appropriately selected patients [1,2].

Over recent years, EBV placement has emerged as a minimally invasive alternative to surgical lung volume reduction, supported by evidence demonstrating improvements in lung function, exercise tolerance, and quality of life. Careful patient selection, including the assessment of collateral ventilation, is crucial to optimize outcomes [1-4].

Although EBVs are generally considered safe, several complications have been described, including pneumothorax, valve migration, hemoptysis, and infection. Pneumothorax is the most common adverse event, with reported incidence rates ranging from 15% to 30% in clinical trials and real-world cohorts, and typically occurs ipsilateral to the treated lung as a consequence of abrupt changes in lung mechanics after valve deployment [5,6].

Patients with heterogeneous emphysema distribution, characterized by marked regional differences in lung destruction and compliance, appear to be at higher risk [5-8], likely due to pronounced regional differences in compliance and stress redistribution, which may also contribute to the rare occurrence of bilateral pneumothorax described in isolated cases [5,6,9]. This phenomenon is primarily driven by the rapid expansion of the remaining lung parenchyma to compensate for the volume loss of the atelectatic segment or lobe, resulting in increased regional stress, alveolar shear forces, and structural failure at the interface between lung units with differing compliance [5,6]. Bilateral tension pneumothorax is an exceedingly rare and potentially life-threatening complication, with scarce documentation in the literature [5-7].

Here, we report a case of bilateral tension pneumothorax following unilateral EBV placement for severe emphysema with hyperinflation, underscoring important diagnostic and management considerations.

## Case presentation

A 61-year-old man with severe emphysema and hyperinflation was admitted to the intensive care unit (ICU) for post-procedural monitoring following unilateral endobronchial valve placement in the right upper lobe (segments B1, B2, and B3). The procedure was uneventful, with no immediate complications observed. At ICU admission, the patient was breathing spontaneously and was not receiving mechanical ventilation.

On day 2 of ICU admission, the patient developed sudden-onset severe respiratory distress, characterized by agitation, profound hypoxemia with oxygen saturation dropping to 65%, central cyanosis, use of accessory respiratory muscles, and bilateral absence of breath sounds on auscultation. He was hemodynamically unstable, presenting with sinus tachycardia (140 beats per minute), severe hypertension (220/110 mmHg), and marked jugular venous distension.

**Figure 1:**
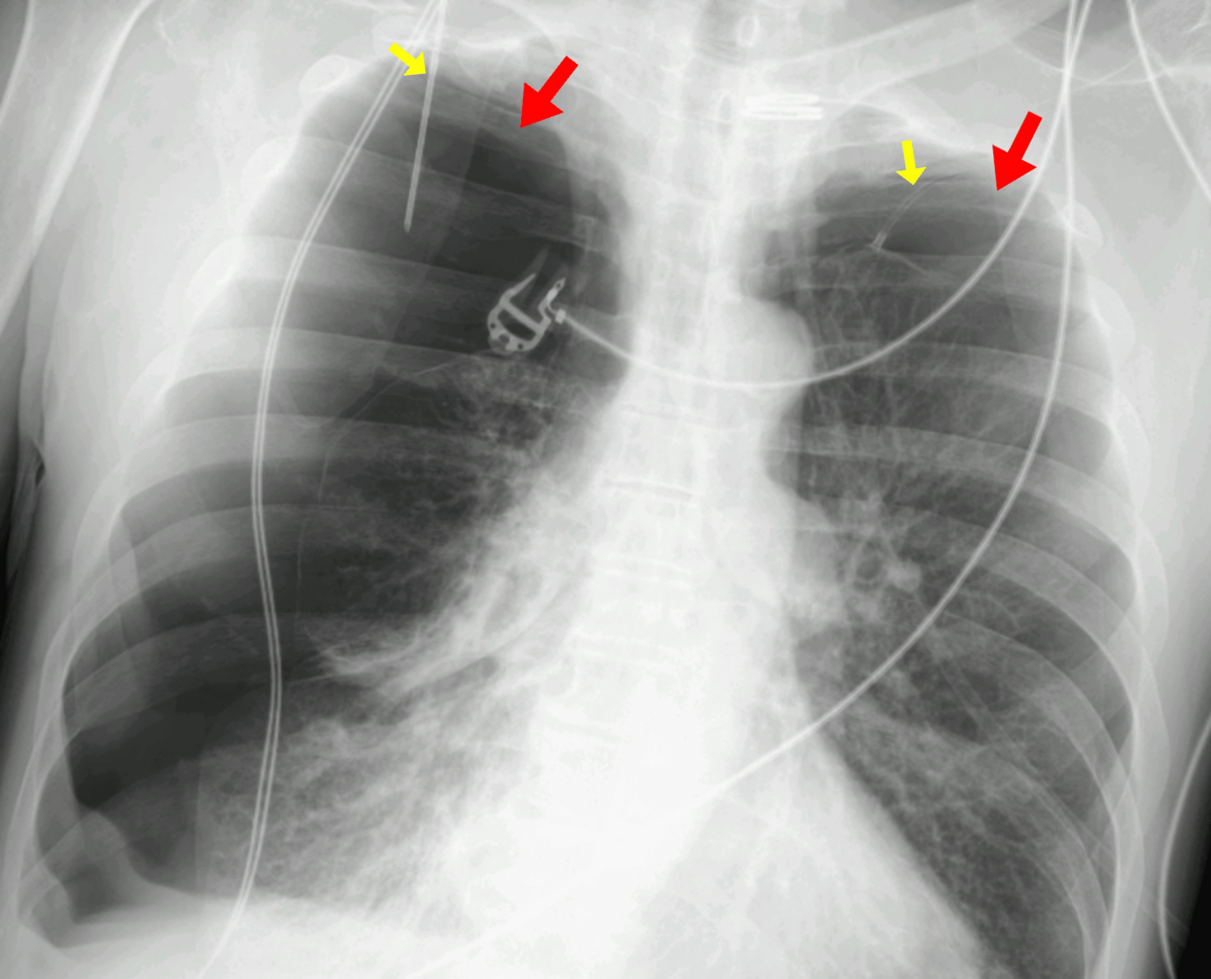
Chest radiograph demonstrating bilateral tension pneumothoraces following unilateral endobronchial valve placement Chest radiograph obtained after emergency bilateral needle decompression demonstrated bilateral pneumothoraces (red arrows), more pronounced on the right side, corresponding to the lung treated with endobronchial valve placement, with associated lung collapse. Needle decompression catheters are visible bilaterally (yellow arrows).

Given the severity of the presentation, a rapid bedside thoracic ultrasound was performed during equipment preparation, revealing bilateral absence of lung sliding and supporting the diagnosis of bilateral tension pneumothorax. Bilateral needle decompression was then performed without delay, with audible evacuation of pressurized air and partial improvement in ventilation. A subsequent chest radiograph confirmed the presence of bilateral pneumothoraces, more pronounced on the right side, corresponding to the lung treated with endobronchial valve placement (Figure 1).

**Figure 2:**
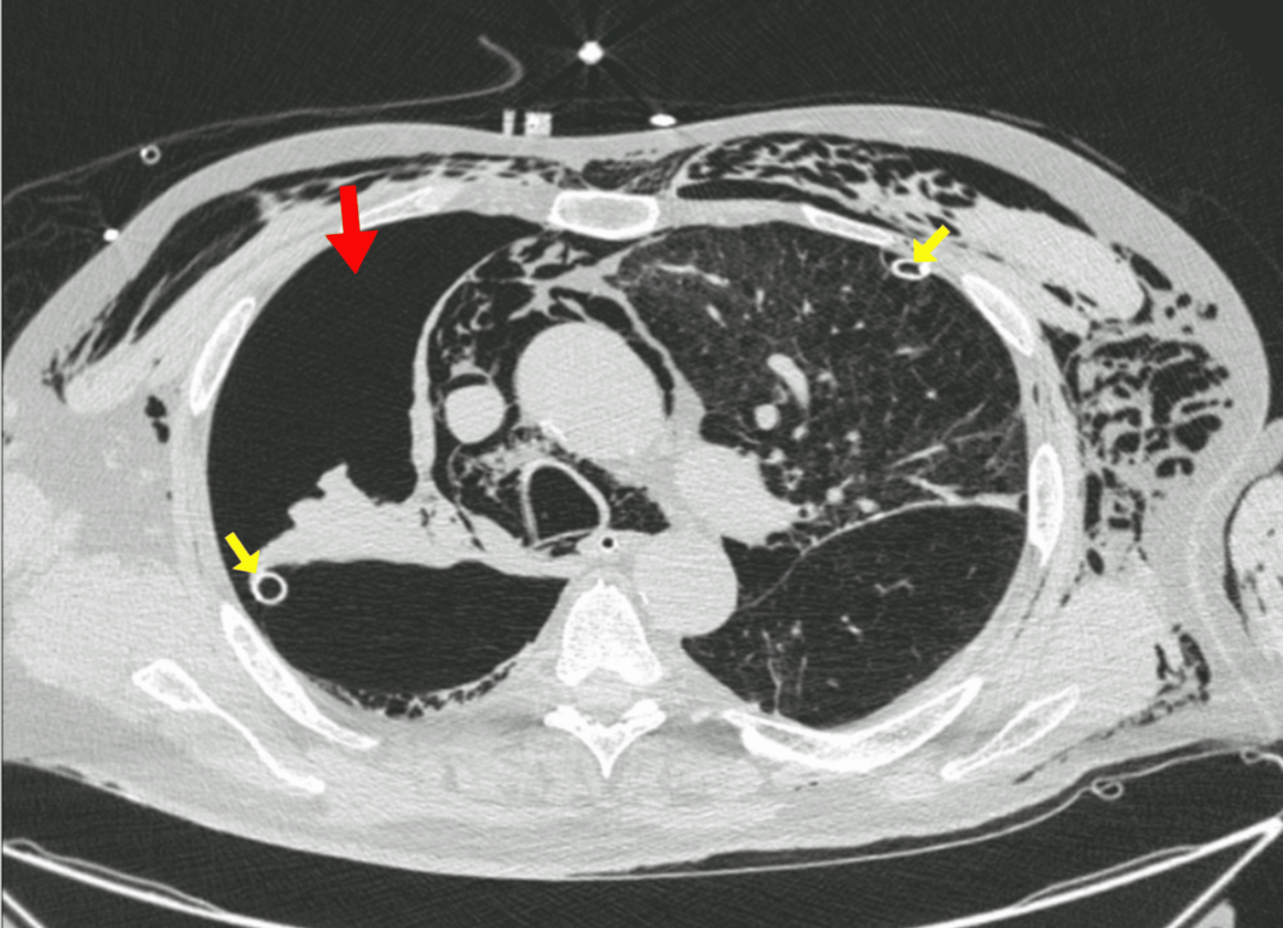
Axial chest computed tomography showing persistent right-sided tension pneumothorax after chest tube placement Axial chest computed tomography showing a persistent large right-sided pneumothorax (red arrow) with incomplete lung re-expansion despite chest tube drainage (yellow arrows), corresponding to the lung treated with endobronchial valve placement.

During preparation for definitive chest drainage, the patient deteriorated rapidly, developing hypotension that progressed to cardiac arrest with pulseless electrical activity. Bilateral chest tubes were promptly inserted, resulting in return of spontaneous circulation after two cycles of advanced life support. Follow-up imaging demonstrated full expansion of the left lung but persistence of a large right-sided pneumothorax (Figure 2), prompting placement of an additional right-sided chest tube in a more anterior position.

Despite five days of spontaneous ventilation, a persistent right-sided pleural air leak of approximately 5-6 L/min was observed, objectively quantified using a digital chest drainage system. In light of the ongoing air leak and concern for a bronchopleural fistula, the endobronchial valves were removed, and chemical pleurodesis with talc was performed, leading to complete resolution of the pneumothorax. The patient had a favorable clinical course and was discharged without neurological deficits.

## Discussion

EV placement is an established bronchoscopic intervention for selected patients with severe emphysema and hyperinflation, offering a minimally invasive alternative to surgical lung volume reduction [1-4]. Pneumothorax is a well-recognized complication of this procedure and is generally attributed to abrupt post-procedural changes in lung mechanics, particularly the rapid expansion of untreated lung regions following lobar atelectasis [5]. This expansion generates increased regional stress and alveolar shear forces at interfaces between lung units with differing compliance, predisposing to alveolar rupture and air leakage [5,6].

In most cases, pneumothorax after EV placement occurs ipsilateral to the treated lung and is detected early during post-procedural monitoring [3-6]. In contrast, bilateral tension pneumothorax is exceptionally rare and represents a life-threatening emergency. The bilateral nature of the pneumothoraces in the present case likely reflects marked mechanical heterogeneity within the emphysematous lungs. Extreme stress redistribution may have affected both lungs despite unilateral valve deployment, particularly in the setting of severe hyperinflation and limited pulmonary reserve [5,6,8,9].

This case highlights the diagnostic challenges posed by bilateral tension pneumothorax, in which classical clinical signs may be subtle or misleading. Although hypotension is more typically associated with tension pneumothorax, the initial presentation in this case was marked by severe hypertension, likely reflecting an early sympathetic response to acute respiratory distress and hypoxemia, prior to rapid cardiovascular collapse. In our patient, the combination of sudden respiratory collapse, jugular venous distension, and bilateral absence of breath sounds raised immediate suspicion. The adjunctive use of bedside thoracic ultrasound during equipment preparation rapidly confirmed bilateral absence of lung sliding, facilitating timely bilateral decompression. This underscores the value of point-of-care ultrasound as a rapid diagnostic adjunct in critically ill patients, particularly when bilateral pathology is suspected and delays in intervention may be fatal [10,11].

Management of pneumothorax following EV placement is typically conservative, with chest drainage sufficing in most cases [3,5,6]. However, persistent air leaks may necessitate valve removal, particularly when a high-output bronchopleural fistula is suspected [5-8]. In this patient, the persistence of a significant air leak despite five days of spontaneous ventilation justified valve removal and chemical pleurodesis, ultimately resulting in complete resolution of the pneumothorax, in line with previously proposed management algorithms [5]. The use of digital air leak quantification helped objectify leak severity and supported the decision for valve removal. Importantly, the absence of neurological sequelae despite cardiac arrest underscores the impact of rapid diagnosis and decisive intervention.

While pneumothorax is a recognized complication of EV placement, to our knowledge, reports of bilateral tension pneumothorax following unilateral EV placement remain exceedingly scarce [5-7] and may therefore be underrecognized in patients with severe emphysema and marked mechanical heterogeneity. This case reinforces the need for heightened clinical vigilance during post-procedural monitoring, even beyond the immediate post-intervention period, and supports the early integration of bedside thoracic ultrasound into the diagnostic pathway for patients with acute respiratory or hemodynamic deterioration after EV therapy.

## Conclusions

Bilateral tension pneumothorax is an exceedingly rare but potentially fatal complication following unilateral EV placement for severe emphysema with hyperinflation. This case highlights the importance of maintaining a high index of suspicion in patients who develop acute respiratory or hemodynamic deterioration after the procedure, even beyond the immediate post-interventional period. Rapid clinical assessment, supported by bedside thoracic ultrasound, may facilitate timely diagnosis and life-saving intervention. Persistent air leaks may require valve removal and pleurodesis, underscoring the need for individualized management and vigilant post-procedural monitoring, while also highlighting the need for further research to better define risk stratification and surveillance strategies in this high-risk population.
